# Supplementary material for: An interoperable web-based platform to support health surveillance against latent tuberculosis infection in health care workers and students: The evolution of CROSSWORD study protocol
Source: PLoS One. 2025 Mar 25;20(3):e0319568. doi: 10.1371/journal.pone.0319568 (PMC11936157; doi:10.1371/journal.pone.0319568)
Supplement: Attachment — Submitted filename: Response to Reviewers.docx [file pone.0319568.s002.docx]

October, 10^th^ 2024

To Editor and Reviewers

PLOS ONE

We would like to greatly thank the Editor and Reviewers who encouraged a revision of the manuscript.

Please find enclosed the revised version (vers. 2) of the Study Protocol article entitled “Protocol the Evolution of CROSSWORD Study: Implementing an interoperable web-based platform to support health surveillance against latent tuberculosis infection in health care workers and students to define prevention strategies and interventions” by Angela Rizzi, Eleonora Nucera, Walter Mazzucco, Pierpaolo Palumbo, Domenico Staiti, Umberto Moscato, Francesco Maria De Simone, Michela Sali, Luca Boldrini, Nikola Dino Capocchiano, Stefano Patarnello, Gabriele Rumi, Raffaella Chini, Valentina Carusi, Michele Centrone, Alessia Di Rienzo, David Longhino, Chiara Laface, Sabato Mellone, Carmelo Massimo Maida, Emanuele Cannizzaro, Luigi Cirrincione, Maria Gabriella Verso, Annalisa Saracino, Francesco Di Gennaro, Luigi Vimercati, Luigi De Maria, Stefania Sponselli, Giancarlo Scoppettuolo, Roberta Pastorino, Antonio Gasbarrini and Riccardo Inchingolo.

PONE-D-24-34897

Protocol the Evolution of CROSSWORD Study: Implementing an interoperable web-based platform to support health surveillance against latent tuberculosis infection in health care workers and students to define prevention strategies and interventions

PLOS ONE

ACADEMIC EDITOR:

Dear Authors,

the manuscript is interesting, but it needs some changes. Both reviewers suggested some changes to be made that I think are appropriate. Finally, I suggest providing more details in the sample size section, describing how the percentages were identified, and the sample size formula used.

**We thank the Academic Editor for the comment. We reviewed and reported the formula adopted for the sample size based on both the first and the second outcomes. We modified the manuscript accordingly. Lines: 222-240.**

Journal Requirements:

When submitting your revision, we need you to address these additional requirements.

1. Please ensure that your manuscript meets PLOS ONE's style requirements, including those for file naming.

**We thank for the comment. The manuscript was modified accordingly.**

2. We note that you have selected “Clinical Trial” as your article type. PLOS ONE requires that all clinical trials are registered in an appropriate registry. Please state the name of the registry and the registration number (e.g. ISRCTN or ClinicalTrials.gov) in the submission data and on the title page of your manuscript.

**We thank for the comment. The manuscript was modified accordingly. Lines: 89-90 and 348-349.**

a) Please provide the complete date range for participant recruitment and follow-up in the methods section of your manuscript.

**We thank for the comment. The manuscript was modified accordingly. Lines: 361-364.**

b) If you have not yet registered your trial in an appropriate registry, we now require you to do so and will need confirmation of the trial registry number before we can pass your paper to the next stage of review. Please include in the Methods section of your paper your reasons for not registering this study before enrolment of participants started. Please confirm that all related trials are registered by stating: “The authors confirm that all ongoing and related trials for this drug/intervention are registered”.

**We thank for the comment. The trial was registered on ClinicalTrial.gov before submission of the first version of the manuscript. We added dedicated sentence in the section “Ethical Considerations and declarations”. Lines: 348-349.**

3. Thank you for stating the following financial disclosure:

“This research was co-funded by the Italian Complementary National Plan PNC-I.1 "Research initiatives for innovative technologies and pathways in the health and welfare sector” D.D. 931 of 06/06/2022, "DARE - DigitAl lifelong pRevEntion" initiative, code PNC0000002.”

Please state what role the funders took in the study. If the funders had no role, please state: "The funders had no role in study design, data collection and analysis, decision to publish, or preparation of the manuscript."

**We thank for the comment. We added dedicated sentence in the section “Funding”. Lines: 65-66.**

4. We note that you have indicated that there are restrictions to data sharing for this study. PLOS only allows data to be available upon request if there are legal or ethical restrictions on sharing data publicly. For more information on unacceptable data access restrictions, please see http://journals.plos.org/plosone/s/data-availability#loc-unacceptable-data-access-restrictions.

Before we proceed with your manuscript, please address the following prompts:

a) If there are ethical or legal restrictions on sharing a de-identified data set, please explain them in detail (e.g., data contain potentially identifying or sensitive patient information, data are owned by a third-party organization, etc.) and who has imposed them (e.g., a Research Ethics Committee or Institutional Review Board, etc.). Please also provide contact information for a data access committee, ethics committee, or other institutional body to which data requests may be sent.

b) If there are no restrictions, please upload the minimal anonymized data set necessary to replicate your study findings to a stable, public repository and provide us with the relevant URLs, DOIs, or accession numbers. For a list of recommended repositories, please see

https://journals.plos.org/plosone/s/recommended-repositories. You also have the option of uploading the data as Supporting Information files, but we would recommend depositing data directly to a data repository if possible.

We will update your Data Availability statement on your behalf to reflect the information you provide.

**We thank for the comment. The minimal anonymized data set necessary to replicate study findings is openly available from BioStudies, S-BSST1682 (https://www.ebi.ac.uk/biostudies/studies/S-BSST1682. DOI: 10.6019/S-BSST1682). Lines: 72-73.**

5. Please include a caption for figures 2 and 3.

**We thank for the comment. We left the first figure as a graphical abstract, if possible, according to editorial guidelines, and added captions for the other 2 figures. Lines: 412-420.**

6. Please include captions for your Supporting Information files at the end of your manuscript, and update any in-text citations to match accordingly. Please see our Supporting Information guidelines for more information: http://journals.plos.org/plosone/s/supporting-information.

**We thank for the comment. The manuscript was modified accordingly. Lines: 409-420.**

Reviewers' comments:

Reviewer #1: The protocol titled "Protocol the Evolution of CROSSWORD Study: Implementing an interoperable web-based platform to support health surveillance against latent tuberculosis infection in healthcare workers and students to define prevention strategies and interventions" is well-structured and addresses a crucial area in tuberculosis (TB) prevention. This study has the potential to make a significant impact on the development of TB preventive strategies in low-TB-burden countries, especially for healthcare workers (HCWs) and students. However, some aspects need clarification and further elaboration:

1. Methods for LTBI Detection Tests:

The manuscript did not clearly define the methods for latent tuberculosis infection (LTBI) detection tests. Both Interferon-Gamma Release Assay (IGRA) and Tuberculin Skin Test (TST) were mentioned in determining the primary endpoint. It is essential to specify whether the IGRA or TST will be used. Since the study involves the conversion of TST, multiple tests are assumed to be done. Information on how often follow-up testing will occur is needed to provide a comprehensive understanding of the testing process.

**We thank the Reviewer for the comment. The diagnosis of LTBI requires both conversion to tuberculin after a documented negative tuberculin skin test (TST) at baseline and subsequent positive QuantiFERON-TB Gold test result performed on the same day as the positive TST result. The manuscript was modified accordingly. Lines: 256-262.**

2. Interaction with the Developed Web-Based Framework:

The article mentions the development of a web-based interactive platform but does not elaborate on how this framework will engage in the prediction and detection of LTBI. It would be helpful to describe how the platform will interact with collected data, monitor participants, or offer decision support for LTBI prediction.

**We thank the Reviewer for the comment. We modified the section “Statistical Analysis” describing the enabling technologies that the research team is adopting for the purposes of the project and mentioning the web-based platform that will be developed as final deliverable of the project. Lines: 332-337.**

3. Rationale for the Estimated Prevalence:

The protocol estimates an LTBI prevalence of 1% in the participating institutes. However, the rationale behind this estimate is not sufficiently explained. Is it derived from the ongoing CROSSWORD study? Clarifying how this estimate was derived, considering factors such as the previous prevalence rates in similar settings or literature, would strengthen the study's methodology.

**We thank the Reviewer for the comment. The assumed prevalence of LTBI of 1% was based on both the prevalence rate of LTBI (0.62%) among healthcare students and postgraduates described by Verso et al. [Ref. 26] and a reasonable estimate derived from the LTBI prevalence (2.1%) described by Coppeta et al. [Ref. 29] in a population of Italian HCWs in Rome. The manuscript was modified accordingly. Lines: 222-226.**

4. Consideration of Changes in Participants’ Roles Over Time:

Since the study involves a potentially prolonged follow-up period, there is a high likelihood that participants’ roles and exposure risks may change (e.g., from students to practicing HCWs). This factor should be taken into account when identifying risk factors and analysing LTBI risk. Clarifying how these changes will be tracked and integrated into the analysis is crucial for ensuring accurate risk assessment.

**We thank the Reviewer for the comment. The section “Statistical Analysis” was modified accordingly. Lines: 328-330.**

Overall Recommendations:

The study protocol is valuable and has the potential to contribute significantly to TB prevention efforts. Addressing these points will enhance the clarity and comprehensiveness of the study. The authors are encouraged to provide detailed explanations regarding the LTBI detection protocols, web-based framework interaction, rationale for prevalence estimation, and adjustments for role changes during the study follow-up.

**We thank the Reviewer for the comment. The manuscript was modified accordingly.**

Reviewer #2: The authors describe the protocol of a longitudinal study (retrospective and prospective), aimed at providing a large amount of data on the incidence and prevalence of latent tuberculosis infection, on the incidence of tuberculosis and on the results of screening tests among health workers and students enrolled in 3 university hospitals in Central and Southern Italy. The objective would be achieved through the digital registration of some selected variables reported in a self-administered questionnaire. The objective is ambitious, and the study protocol could validly contribute to scientific progress on a pathology present throughout the world, slightly increasing after the pandemic and with a significant socioeconomic burden even in low-incidence countries, such as Italy. Moreover, university students, an understudied population strata, are included.

However, I think that the submitted description of the protocol should be enriched with details and few methodological aspects should be further clarify. I recommend substantial revisions that take into account the considerations reported below.

**We thank the Reviewer for the comment.**

First of all, it's necessary to know how many employees and students are in the 3 hospitals considered, this to have information on the feasibility of reaching the sample size in the expected period. It might be useful to know how many people are divided into different age groups, how many students, whether only medical courses or other types of courses, such as engineering, are included, how many men and how many women. It might also be useful to know what is the incidence of tuberculosis in the reference population of the 3 hospitals considered and what is the estimated incidence of active or latent tuberculosis in previous periods among the healthcare workers and students. As regards the feasibility, add information on the progress of the Crossword study, which represents the retrospective phase of the study protocol. Moreover, it's necessary to provide more details on which aspects of the Periskope-TB study were captured in the described protocol, to better support the choice of selected variables.

**We thank the Reviewer for the comment. We specified the potential involvement of all employees and students attending medical, specialization and health professions degree courses in the 3 university hospital centers involved in the project making it possible to reach the target population. We modified the section “The sample size”. Lines: 222-240.**

**Regarding the incidence of tuberculosis in the reference population of the 3 hospitals considered, we have published data on prevalence rate of LTBI (0.62%) among healthcare students and postgraduates described by Verso et al. [Ref. 26]. Therefore, we assumed a reasonable estimate derived also from the LTBI prevalence (2.1%) described by Coppeta et al. [Ref. 29] in a population of Italian HCWs in Rome. The manuscript was modified accordingly. Lines: 222-225.**

**Regarding the retrospective phase of the study, the final data analysis will be completed by November 2024. We added this detail in the manuscript. Lines: 360-361.**

**Finally, we specified the predictors, reported by Gupta et al. [Ref. 4] in the final version of prognostic model described in PERISKOPE-TB, included in our questionnaire. We modified the manuscript accordingly. Lines: 179-181.**

The DARE project is mentioned but its contribution to this protocol is unclear. More information on the Dare study (bibliographic citation, if available) could clarify how the predictive algorithms adopted in this protocol were digitized and created.

**We thank the Reviewer for the comment. We added details on DARE Project and reference n° 28. Lines: 200-205.**

It's necessary to stratify the sample size calculation by workers and students, sex and age groups, considering that the enrolled population includes workers and students probably in a wide age range.

Since workers and students are subjected to annual health surveillance, it is not clear whether they will answer only once or multiple times (and how many?) to the proposed questionnaire.

**We thank the Reviewer for the comment. We reviewed and reported the formula adopted for the sample size based on both the first and the second outcomes. The stratification of sample size calculation in subgroups was not *a priori* planned due to expected impact on the number of subjects to be enrolled, mining the feasibility of the whole project. Lines: 222-240.**

**HCWs and students will respond to the questionnaire survey only once. We modified the manuscript accordingly. Lines: 289-290.**

Authors should consider adding to the exclusion criteria, having had tuberculosis or having already undergone chemoprophylaxis.

**We thank the Reviewer for the comment. We modified the manuscript accordingly. Lines: 248-249.**

About the primary outcomes, consider adding a symptom questionnaire. It should also be clarified how to consider the possible movement between working units. Among the secondary outcomes, it would be better to add a definition of exposure to TB-infected patients, to avoid different descriptions.

**We thank the Reviewer for the comment, but symptom assessment was not included in the study protocol. The possible movement between working units is considered among the list of possible risk factors. The meaning of exposure to an infected patient was clarified. We modified the manuscript accordingly. Lines: 275-276.**

I would like the authors to explain the reasons why they collect health information through a self-administered questionnaire and not through medical records, which would avoid recall bias and inaccuracies.

**We thank the Reviewer for the comment. In the three university hospital centers involved in the project, the questionnaire is proposed and administered to HCWs and students, not to patients, therefore it is not possible to consult medical records, but rather health dossiers that contain the results of clinical evaluations aimed at assessing suitability for work.**

For question 4 I would put an indicative time (1 month or 3 months of stay).

**We thank the Reviewer for the comment. We modified the question adding “for at least 3 months”. See Figure 2.**

Consider adding a table or a figure to list the recorded variables. Line 243: it's not clear if the items listed will be recorded.

**We thank the Reviewer for the comment. We specified that the list of possible risk factors will be recorded. Line: 269.**

Furthermore, authors should reread the text to make the English grammar more fluent. Few typos should be corrected: uniform the acronym TB or TBC, replace the word tuberculosis with the somewhat long acronym in the text; add title and description of figure 2 and 3.

**We thank the Reviewer for the comment. We modified the manuscript accordingly.**

I would encourage the authors to consider making the dataset public, possibly removing variables that could identify the respondent (e.g. department, course of study, etc.).

**We thank for the comment. The minimal anonymized data set necessary to replicate study findings is openly available from BioStudies, S-BSST1682 (https://www.ebi.ac.uk/biostudies/studies/S-BSST1682. DOI: 10.6019/S-BSST1682). Lines: 72-73.**

Authors should check bibliography according to the journal's instructions, in particular citations 1, 5, 6, 7, 9, 19, 20.

**We thank the Reviewer for the comment. We modified the manuscript accordingly.**

With the best regards,

Angela Rizzi, Eleonora Nucera, Walter Mazzucco, Pierpaolo Palumbo, Domenico Staiti, Umberto Moscato, Francesco Maria De Simone, Michela Sali, Luca Boldrini, Nikola Dino Capocchiano, Stefano Patarnello, Gabriele Rumi, Raffaella Chini, Valentina Carusi, Michele Centrone, Alessia Di Rienzo, David Longhino, Chiara Laface, Sabato Mellone, Carmelo Massimo Maida, Emanuele Cannizzaro, Luigi Cirrincione, Maria Gabriella Verso, Annalisa Saracino, Francesco Di Gennaro, Luigi Vimercati, Luigi De Maria, Stefania Sponselli, Giancarlo Scoppettuolo, Roberta Pastorino, Antonio Gasbarrini and Riccardo Inchingolo.

Corresponding Author:

Riccardo Inchingolo, MD, PhD

UOC Pneumologia, Fondazione Policlinico Universitario A. Gemelli IRCCS. Largo A. Gemelli, 8 – 00168 – Rome, Italy.

riccardo.inchingolo@policlinicogemelli.it

Corresponding Author will receive all editorial communications

The authors declare that the manuscript, or specified parts of it, have not been and will not be submitted elsewhere for publication.
