# Supplementary material for: An interoperable web-based platform to support health surveillance against latent tuberculosis infection in health care workers and students: The evolution of CROSSWORD study protocol
Source: PLoS One. 2025 Mar 25;20(3):e0319568. doi: 10.1371/journal.pone.0319568 (PMC11936157; doi:10.1371/journal.pone.0319568)
Supplement: Attachment — Submitted filename: Response_to_Reviewers_auresp_2.docx [file pone.0319568.s003.docx]

January, 15^th^ 2025

To Editor and Reviewers

PLOS ONE

We would like to greatly thank the Editor and Reviewers who encouraged a further revision of the manuscript.

Please find enclosed the revised version (vers. 3) of the Study Protocol article entitled “Protocol the Evolution of CROSSWORD Study: Implementing an interoperable web-based platform to support health surveillance against latent tuberculosis infection in health care workers and students to define prevention strategies and interventions” by Angela Rizzi, Eleonora Nucera, Walter Mazzucco, Pierpaolo Palumbo, Domenico Staiti, Umberto Moscato, Francesco Maria De Simone, Michela Sali, Luca Boldrini, Nikola Dino Capocchiano, Stefano Patarnello, Gabriele Rumi, Raffaella Chini, Valentina Carusi, Michele Centrone, Alessia Di Rienzo, David Longhino, Chiara Laface, Sabato Mellone, Carmelo Massimo Maida, Emanuele Cannizzaro, Luigi Cirrincione, Maria Gabriella Verso, Annalisa Saracino, Francesco Di Gennaro, Luigi Vimercati, Luigi De Maria, Stefania Sponselli, Giancarlo Scoppettuolo, Roberta Pastorino, Antonio Gasbarrini and Riccardo Inchingolo.

PONE-D-24-34897

Protocol the Evolution of CROSSWORD Study: Implementing an interoperable web-based platform to support health surveillance against latent tuberculosis infection in health care workers and students to define prevention strategies and interventions

PLOS ONE

ACADEMIC EDITOR:

Dear Authors,

your manuscript has been greatly improved, and both reviewers agree on minor revisions. Please submit the revision by the deadline and provide a general check of the English language.

**We thank the Academic Editor for the comment. We modified the manuscript accordingly.**

Reviewers' comments:

Reviewer #1: The author described the primary outcome as prevalence of LTBI. The LTBI was defined as positive TST plus positive QuantiFERON for participants with a baseline negative TST. Will participants with a baseline positive TST be excluded from the cohort? Base one such definition, the primary outcome seems to be the incidence of TB infection instead of prevalence of TB infection.

2. How many TST tests will be done for each participant? When will a follow-up TST test be done for a participant?

**We thank the Reviewer for the comment. We detailed the definition of the primary outcome including both the evidence of positive results of TST at enrollment and tuberculin conversion defined as negative TST before enrollment and positive at enrollment. All criteria are verified at enrollment due to cross-sectional nature of the study protocol. Furthermore, we added the exclusion criterion of positive TST performed within 12 months prior to enrollment to exclude subjects from the cohort and specified the number of scheduled TST for each subject. Finally, we specified that the study protocol does not provide follow-up for research purposes, however, each participant continues regular annual follow-up as per routine clinical practice. Lines: 249-265.**

Reviewer #3: Management of latent tuberculosis infection (LTBI) is a key component of any TB elimination strategy, as people with LTBI represent a large "human reservoir" for the disease. In these purposes the European Centre for Disease Prevention and Control (ECDC), provides evidence-based guidance for the implementation of public health programmes for the management of LTBI in the European Union (EU) and the European Economic Area (EEA), which includes, beyond the prevalence of the LBT, the identification of risk groups. In this context, the present article fits well.

The authors made most of the changes requested by the reviewer 2; in the references the 7 must be corrected. In addition, if possible, choose for publication a title more impactful, which is currently too long.

**We thank the Reviewer for the comment. We removed reference n°7 from the list due to inability to access. Finally, we modified the title as required. Lines: 4-6.**

With the best regards,

Angela Rizzi, Eleonora Nucera, Walter Mazzucco, Pierpaolo Palumbo, Domenico Staiti, Umberto Moscato, Francesco Maria De Simone, Michela Sali, Luca Boldrini, Nikola Dino Capocchiano, Stefano Patarnello, Gabriele Rumi, Raffaella Chini, Valentina Carusi, Michele Centrone, Alessia Di Rienzo, David Longhino, Chiara Laface, Sabato Mellone, Carmelo Massimo Maida, Emanuele Cannizzaro, Luigi Cirrincione, Maria Gabriella Verso, Annalisa Saracino, Francesco Di Gennaro, Luigi Vimercati, Luigi De Maria, Stefania Sponselli, Giancarlo Scoppettuolo, Roberta Pastorino, Antonio Gasbarrini and Riccardo Inchingolo.

Corresponding Author:

Riccardo Inchingolo, MD, PhD

UOC Pneumologia, Fondazione Policlinico Universitario A. Gemelli IRCCS. Largo A. Gemelli, 8 – 00168 – Rome, Italy.

riccardo.inchingolo@policlinicogemelli.it

Corresponding Author will receive all editorial communications

The authors declare that the manuscript, or specified parts of it, have not been and will not be submitted elsewhere for publication.
