# Supplementary material for: An interoperable web-based platform to support health surveillance against latent tuberculosis infection in health care workers and students: The evolution of CROSSWORD study protocol
Source: PLoS One. 2025 Mar 25;20(3):e0319568. doi: 10.1371/journal.pone.0319568 (PMC11936157; doi:10.1371/journal.pone.0319568)
Supplement: Attachment — Submitted filename: Response_to_Reviewers_auresp_3.docx [file pone.0319568.s004.docx]

February, 1^st^ 2025

To Editor and Reviewers

PLOS ONE

We would like to greatly thank the Editor and Reviewers who encouraged a further revision of the manuscript.

Please find enclosed the revised version (vers. 4) of the Study Protocol article entitled “An interoperable web-based platform to support health surveillance against latent tuberculosis infection in health care workers and students: the Evolution of CROSSWORD Study Protocol” by Angela Rizzi, Eleonora Nucera, Walter Mazzucco, Pierpaolo Palumbo, Domenico Staiti, Umberto Moscato, Francesco Maria De Simone, Michela Sali, Luca Boldrini, Nikola Dino Capocchiano, Stefano Patarnello, Gabriele Rumi, Raffaella Chini, Valentina Carusi, Michele Centrone, Alessia Di Rienzo, David Longhino, Chiara Laface, Sabato Mellone, Carmelo Massimo Maida, Emanuele Cannizzaro, Luigi Cirrincione, Maria Gabriella Verso, Annalisa Saracino, Francesco Di Gennaro, Luigi Vimercati, Luigi De Maria, Stefania Sponselli, Giancarlo Scoppettuolo, Roberta Pastorino, Antonio Gasbarrini and Riccardo Inchingolo.

PONE-D-24-34897R2

An interoperable web-based platform to support health surveillance against latent tuberculosis infection in health care workers and students: the Evolution of CROSSWORD Study Protocol

PLOS ONE

ACADEMIC EDITOR:

Dear Authors,

Please consider an important final suggestion from Reviewer 1 regarding the timing of the QuantiFERON-TB Gold (QFT) test.

**We thank the Academic Editor for the comment. We modified the manuscript accordingly.**

Reviewers' comments:

Reviewer #1: The author addressed my concern. One minor suggestion:

I appreciate the comprehensive definition of LTBI used in the study; however, I would like to suggest a minor revision regarding the timing of the QuantiFERON-TB Gold (QFT) test. The current definition requires the QFT test to be performed on the same day as the positive TST result. In clinical practice, logistical challenges such as laboratory processing delays, sample collection issues, or the need for repeat blood draws may prevent same-day testing. This strict requirement could lead to unnecessary exclusions due to protocol deviations, even in cases where the QFT result is positive within a reasonable timeframe. To improve the feasibility of the study without compromising diagnostic accuracy, I recommend allowing a reasonable time window for QFT testing rather than requiring it strictly on the same day. This adjustment would better align with real-world clinical workflows and help avoid potential difficulties in data interpretation and patient classification.

**We thank the Reviewer for the comment. To improve the feasibility of the study without compromising diagnostic accuracy, we introduced a reasonable time window of 5days for QFT testing. We modified accordingly. Lines: 257-258.**

Reviewer #3: The authors made the changes required and now I believe that the article can be accepted.

**We thank the Reviewer for the comment.**

With the best regards,

Angela Rizzi, Eleonora Nucera, Walter Mazzucco, Pierpaolo Palumbo, Domenico Staiti, Umberto Moscato, Francesco Maria De Simone, Michela Sali, Luca Boldrini, Nikola Dino Capocchiano, Stefano Patarnello, Gabriele Rumi, Raffaella Chini, Valentina Carusi, Michele Centrone, Alessia Di Rienzo, David Longhino, Chiara Laface, Sabato Mellone, Carmelo Massimo Maida, Emanuele Cannizzaro, Luigi Cirrincione, Maria Gabriella Verso, Annalisa Saracino, Francesco Di Gennaro, Luigi Vimercati, Luigi De Maria, Stefania Sponselli, Giancarlo Scoppettuolo, Roberta Pastorino, Antonio Gasbarrini and Riccardo Inchingolo.

Corresponding Author:

Riccardo Inchingolo, MD, PhD

UOC Pneumologia, Fondazione Policlinico Universitario A. Gemelli IRCCS. Largo A. Gemelli, 8 – 00168 – Rome, Italy.

riccardo.inchingolo@policlinicogemelli.it

Corresponding Author will receive all editorial communications

The authors declare that the manuscript, or specified parts of it, have not been and will not be submitted elsewhere for publication.
